# Supplementary material for: Flax Biomass Conversion via Controlled Oxidation: Facile Tuning of Physicochemical Properties
Source: Bioengineering (Basel). 2020 Apr 27;7(2):38. doi: 10.3390/bioengineering7020038 (PMC7355426; doi:10.3390/bioengineering7020038)
Supplement: Supplementary file 1 [file bioengineering-07-00038-s001.pdf]

Supporting Information

Flax Biomass Conversion via Controlled Oxidation: Facile Tuning of Physicochemical Properties.

Leila Dehabadi[1,2], Abdalla H. Karoyo[1], Majid Solleimani[3], Wahab O. Alabi[4], Carey J. Simonson[4], and Lee D. Wilson[1,*]

1 Department of Chemistry, University of Saskatchewan, 110 Science Place, Saskatoon, SK, S7N 5C9; led082@mail.usask.ca (L. D.); Abdalla.karoyo@usask.ca (A. H. K); lee.wilson@usask.ca (L. D. W.).

2 Dr. Ma's Laboratories Inc. Unit 4, 8118 North Fraser Way, Burnaby, BC, V5J 0E5, Canada

3 Department of Chemical and Biological Engineering, University of Saskatchewan, 57 Campus Drive, Saskatoon, SK, S7N 5A9; mas233@mail.usask.ca

4 Department of Mechanical Engineering, University of Saskatchewan, 57 Campus Drive, Saskatoon, SK, S7N 5A9; woa792@mail.usask.ca; cjs330@mail.usask.ca.

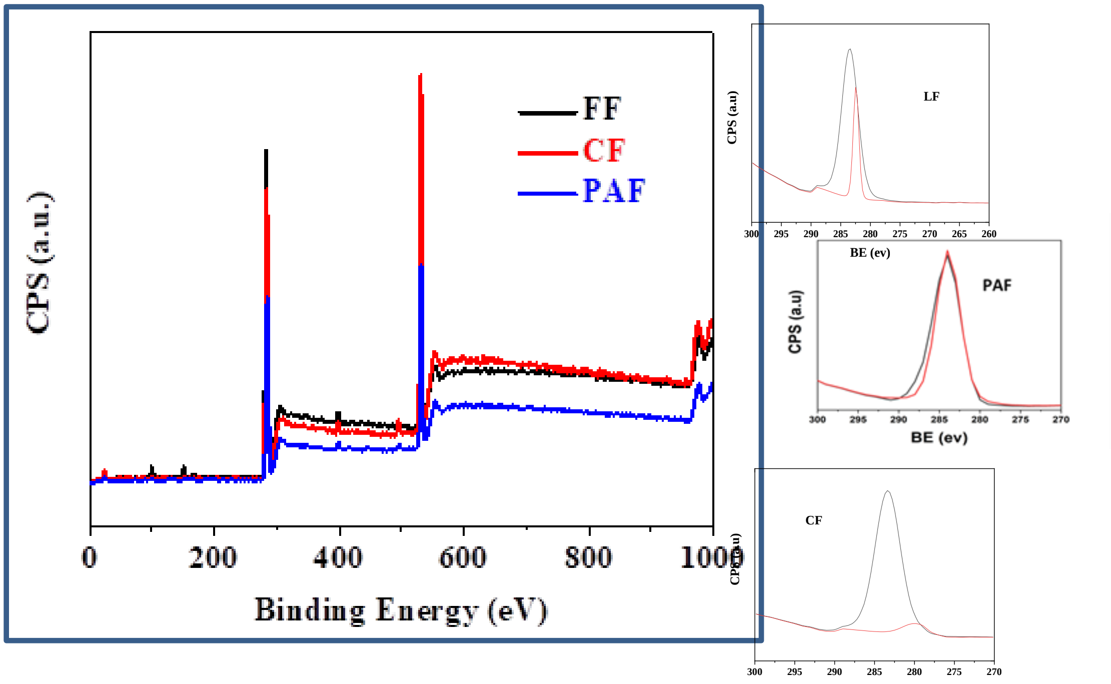

Figure S1. XPS survey and deconvoluted spectra for linen fiber (LF) and its treated forms (CF and PAF).
